# The Use of Biophysical Flow Models in the Surgical Management of Patients Affected by Chronic Thromboembolic Pulmonary Hypertension

**DOI:** 10.3389/fphys.2018.00223

**Published:** 2018-03-13

**Authors:** Martina Spazzapan, Priya Sastry, John Dunning, David Nordsletten, Adelaide de Vecchi

**Affiliations:** ^1^King's College London, GKT School of Medical Education, London, United Kingdom; ^2^Cardiothoracic Surgery Unit, Papworth Hospital NHS Foundation Trust, Cambridge, United Kingdom; ^3^King's College London, School of Biomedical Engineering and Imaging Sciences, St. Thomas' Hospital, London, United Kingdom

**Keywords:** CTEPH, HPC-based computational modeling, biophysical flow modeling, patient specific computational modeling, computational physiology

## Abstract

**Introduction:** Chronic Thromboembolic Pulmonary Hypertension (CTEPH) results from progressive thrombotic occlusion of the pulmonary arteries. It is treated by surgical removal of the occlusion, with success rates depending on the degree of microvascular remodeling. Surgical eligibility is influenced by the contributions of both the thrombus occlusion and microvasculature remodeling to the overall vascular resistance. Assessing this is challenging due to the high inter-individual variability in arterial morphology and physiology. We investigated the potential of patient-specific computational flow modeling to quantify pressure gradients in the pulmonary arteries of CTEPH patients to assist the decision-making process for surgical eligibility.

**Methods:** Detailed segmentations of the pulmonary arteries were created from postoperative chest Computed Tomography scans of three CTEPH patients. A focal stenosis was included in the original geometry to compare the pre- and post-surgical hemodynamics. Three-dimensional flow simulations were performed on each morphology to quantify velocity-dependent pressure changes using a finite element solver coupled to terminal 2-element Windkessel models. In addition to transient flow simulations, a parametric modeling approach based on constant flow simulations is also proposed as faster technique to estimate relative pressure drops through the proximal pulmonary vasculature.

**Results:** An asymmetrical flow split between left and right pulmonary arteries was observed in the stenosed models. Removing the proximal obstruction resulted in a reduction of the right-left pressure imbalance of up to 18%. Changes were also observed in the wall shear stresses and flow topology, where vortices developed in the stenosed model while the non-stenosed retained a helical flow. The predicted pressure gradients from constant flow simulations were consistent with the ones measured in the transient flow simulations.

**Conclusion:** This study provides a proof of concept that patient-specific computational modeling can be used as a noninvasive tool for assisting surgical decisions in CTEPH based on hemodynamics metrics. Our technique enables determination of the proximal relative pressure, which could subsequently be compared to the total pressure drop to determine the degree of distal and proximal vascular resistance. In the longer term this approach has the potential to form the basis for a more quantitative classification system of CTEPH types.

## Introduction

Chronic Thromboembolic Pulmonary Hypertension (CTEPH) is a form of pulmonary hypertension that arises as a complication in patients who suffered an acute embolic event (Pengo et al., 2004). For most patients this progressively fatal disease manifests several months or years following the event. Over this asymptomatic period, thromboembolic material in the pulmonary trunk is incorporated into arterial walls, gradually narrowing the vessel lumen and consequently increasing the peripheral pulmonary vascular resistance (PVR) (McNeil and Dunning, 2007). This raised PVR increases the right heart workload, leading to right ventricular failure. Although a single unresolved event such as the presence of a thrombotic occlusion in one of the pulmonary arteries is usually responsible for the development of CTPEH, in patients with the most severe forms of the disease small vessel arteriopathy with microvasculature remodeling is often observed (McNeil and Dunning, 2007). It is understood that these changes in the peripheral vasculature have an important yet still unclear functional role in further raising PVR (Ruiz-Cano et al., 2015).

While a number of risk factors for CTEPH have been identified, none of these are sufficiently significant to be used in creating scoring criteria, leaving CTEPH diagnosis mostly down to clinical experience and expertise, aided by anatomical knowledge derived from imaging data (Thistlethwaite et al., 2008). Similar limitations are found when planning surgical treatment, i.e., pulmonary thromboendarterectomy (PTE), a complex procedure requiring median sternotomy, cardiopulmonary bypass, and circulatory arrest to remove both the thrombus and the inner layers of the affected artery (Jamieson et al., 2003; Thistlethwaite et al., 2008). This procedure is usually deemed appropriate if the obstruction to the flow is proximal to the segmental branches, i.e., in the main or lobar pulmonary arteries. When the peripheral microvasculature is compromised by the disease progression, increased flow resistance results from both proximal occlusion, and adverse remodeling of inaccessible parts of the pulmonary vasculature and therefore PTE would not necessarily lead to an improvement in PVR (Kim, 2006). As a result, this type of surgery has higher rates of failure in CTEPH patients where peripheral remodeling—and not a proximal stenosis—is the major contributor to pulmonary hypertension (van de Veerdonk et al., 2011). Given the challenges and risks posed by this major procedure, PTE should only be performed if strictly necessary. It is therefore of key importance to determine the relative contribution of peripheral remodeling and proximal occlusion to the increase in PVR in each CTEPH patient, and use this information to derive robust selection criteria for PTE.

Computed Tomography (CT) pulmonary angiography is the gold standard investigation tool for determining both the presence and the extent of CTEPH in common clinical practice (McNeil and Dunning, 2007). Ventilation-perfusion scans are also performed to differentiate between various causes of pulmonary hypertension, including CTEPH. In addition to anatomical evaluation via imaging data, invasive assessment of pulmonary arterial pressure via cardiac catheterization also provides a diagnostic threshold for intervention. Specifically, CTEPH is associated with a mean pulmonary artery pressure above 25 mmHg and a pulmonary capillary wedge pressure of no more than 15 mmHg, in conjunction with the presence of chronic occlusive thrombi (Pepke-Zaba et al., 2011; Lau and Humbert, 2015). More recently the diastolic pressure gradient (DPG), a hemodynamic marker based on the difference between the mean diastolic pulmonary artery pressure and the mean pulmonary capillary wedge pressure, has been proposed as an effective diagnostic index in pulmonary hypertension, with DPG values >7 mmHg indicating adverse remodeling of the pulmonary vasculature (Gerges et al., 2013; Mazimba et al., 2016). The high inter-individual variability of patient morphologies in CTEPH, in conjunction with the progressive nature of the disease and its lack of specific symptoms, means that diagnosis and prognosis must rely on both anatomical and functional evaluations to be effective. However, due to risks associated with catheterization, invasive pressure measurements cannot be performed frequently in CTEPH patients and cardiac imaging only allows for anatomical evaluations, without providing insight into the patient hemodynamics. Further, there are currently no well-defined criteria to differentiate between proximal and distal forms of CTEPH (Galiè et al., 2009).

Such clinical context provides an ideal environment to test the potential of more sophisticated biophysical computational modeling to address the present difficulties in patient selection for PTE (McLaughlin et al., 1998). Anatomically realistic computational models of the arterial system can be tailored to the individual pathophysiology of the pulmonary arteries via patient-specific boundary conditions, providing a personalized description of the disease that is particularly useful in this type of pathologies, where the exclusive use of population-based biomarkers for patient selection results in sub-optimal treatment strategies (Morris et al., 2016). Image-based three-dimensional Computational Fluid Dynamics (CFD) or Fluid-Structure Interaction (FSI) simulations of arterial flow can thus provide a flexible and powerful tool to elucidate the driving mechanism of disease progression (Taylor and Figueroa, 2009). Personalized CFD modeling was applied to assist in treatment planning by quantifying noninvasively hemodynamic parameters such as pressure (Kheyfets et al., 2013) and wall shear stress (WSS) (Tang et al., 2011, 2012). However, despite its clinical potential, image-based modeling in the context of CTEPH is still largely unexplored, with a limited number of studies based on simplified models. The implications of PTE in patients with different relative contribution of distal remodeling and proximal stenosis to the total PVR has been investigated using a simplified mathematical model based on the electrical analogy with two resistors in parallel, representing the proximal and peripheral resistances, respectively (Poullis, 2015). Similarly 1D models that rely on the wave equation and 0D Windkessel models have also been used to characterize pressure noninvasively in pulmonary hypertension patients using Phase Contrast MRI data as input (Lungu et al., 2014). However, these studies do not leverage on the recent progress in 3D personalized CFD modeling that was largely applied to a wide spectrum of cardiovascular diseases (de Zélicourt et al., 2010; Ladisa et al., 2010; Les et al., 2010; Coogan et al., 2011; Cebral et al., 2015; Numata et al., 2016; Arthurs et al., 2017; Youssefi et al., 2017), leaving this clinical question largely unexplored.

In this study, we investigated the potential of personalized CFD simulations of the pulmonary arteries to provide a clinical tool to better understand the role of patient-specific morphology and hemodynamics in determining the major contributor to raised PVR in CTEPH. All the simulations were carried out on realistic, high-resolution anatomical models of the pulmonary arteries by combining High-Performance Computing (HPC) and high-resolution Finite Element Method (FEM) modeling using the software package CHeart, which has been extensively validated and applied to simulate cardiovascular hemodynamics in a wide range of pathologies (de Vecchi et al., 2012, 2014a,b; McCormick et al., 2014; Lee et al., 2016; Hessenthaler et al., 2017). Three CTEPH patients who underwent PTE were modeled as a proof of concept that such an approach can contribute to improve patient selection criteria for PTE, reducing the need for more invasive pressure measurements to inform clinicians on the likely prognosis post-intervention. Special emphasis was placed on how to most accurately and efficiently model pulmonary vasculature in order to obtain the best compromise between anatomical and physiological accuracy, and computational effort. This investigation shows the potential of image-based personalized CFD modeling to support and improve the clinical decision-making process in diseases where “conventional” treatments derived from population-based guidelines are less effective or, in some cases, inadequate. Moreover, demonstrating that patient-specific models can be generated and applied to a specific clinical question without excessive computational demand, both in terms of time and resources, further supports the potential for a targeted clinical applicability of this technology.

## Materials and methods

### Finite-element model generation from patient data

The clinical data for this study was obtained from Royal Papworth Hospital (Cambridge, UK) from three CTEPH patients who had undergone PTE. Patient 1 and 3 presented a stenosis on the right pulmonary artery (RPA), while in Patient 2 the partial occlusion was located on the left pulmonary artery (LPA). Further, in Patient 3 a large portion of the right lung had been surgically removed in a previous intervention. The study was approved by the local ethics review committees and all patients had given written consent.

**Figure 1:**
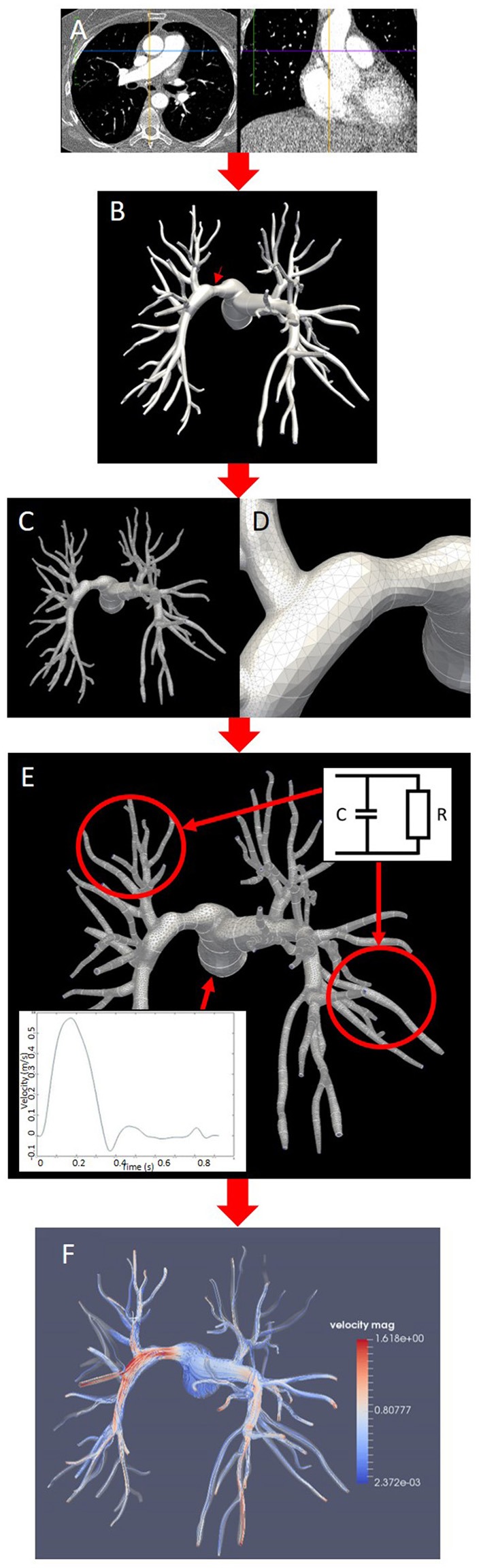
Pipeline for the generation of patient-specific models and simulations in this study (Patient 2, posterior view). **(A)** Post-surgical CT pulmonary angiography. **(B)** Segmentation of the stenosed model with surface interpolation and arrow indicating stenosis on the LPA. **(C,D)** tetrahedral mesh **(C)** with curvature refinement **(D)**. **(E)** Prescription of boundary conditions at the outlets (two-element Windkessel model) and at the inlet (Dirichlet condition on flow velocity); **(F)** CFD simulation of blood flow visualized using streamlines colored by velocity magnitude.

The modeling workflow for the generation and personalization of the image-based models is illustrated in Figure 1. All models in this study were made patient-specific using anatomical information extracted from CT pulmonary angiography (CTPA) data (Figure 1A). All CTPA scans were performed using a combined chest dual energy (140–80 kV) acquisition with 1 mm slice thickness on a high-resolution Siemens Somatom Force scanner. The morphological models of the pulmonary arteries of each patient were initially based on the post-operative scans. Each morphology was segmented using the semi-automatic segmentation tool from the software package CRIMSON (Cardiovascular Integrated Modeling and SimulatiON), which was previously validated and used to simulate hemodynamics in a variety of cardiovascular problems (Lau and Figueroa, 2015; Arthurs et al., 2016, 2017; Khlebnikov and Figueroa, 2016; CRIMSON, 2017). The segmentation technique relies on the definition of paths along the centerline of each vessel, followed by the manual segmentation of the vessel cross sections at multiple locations along the centerline. The contours are then interpolated to produce smooth NURBS surfaces that approximate the vessel wall (Figure 1B). Interlobar arteries were segmented from the main pulmonary artery (MPA), as well as sub-segmental trunks until the third generation. Table 1 reports the number of outlets segmented from each morphology, as well as information about the inlet and outlet surface areas and mesh size of each model.

**Table 1:** Anatomical parameters and mesh size of the segmented morphologies.

**Patient**	**Inlet surface area (mm^2^)**	**LPA total outlet surface area (mm^2^)**	**RPA total outlet surface area (mm^2^)**	**Number of outlets in the model**	**Mesh size (tetrahedral elements)**
Patient 1	722.88	264.90	168.72	31 LPA, 29 RPA	3,468,196
Patient 2	550.24	131.90	105.36	29 LPA, 31 RPA	3,377,433
Patient 3	613.28	105.16	40.55	31 LPA, 12 RPA	2,812,081

*LPA, left pulmonary artery; RPA, right pulmonary artery*.

From these segmentations, tetrahedral volume meshes with 2.8–3.4 million elements were generated with minimum and maximum edge length over the whole meshes of 5.9 and 0.0085 mm, respectively. Regional curvature refinement was applied to critical areas such as bifurcations and sudden changes in the vessel diameter to ensure numerical accuracy in the flow simulations (Figure 1D). A boundary layer made of three concentric layers of refined tetrahedral elements, with total thickness of 1 mm, was also added near the wall to resolve near-wall boundary layers. The effect of stenosis in the pulmonary arteries was investigated by manipulating the post-operative model to introduce a local narrowing in the proximal pulmonary vessels (Figure 1E). Each original segmentation was therefore modified to include a focal stenosis upstream of lobar divisions, while all other contours were left untouched and the models were subsequently meshed. The stenosis severity was then calculated as the percentage reduction in diameter, i.e., the ratio of the difference between the original and the stenotic diameter over the original diameter. Patient 1 had a percentage diameter reduction of 43.8% on the RPA, Patient 2 of 40.3% on the LPA. The mesh from Patient 3 was first modified to add a 37.1% diameter reduction (Patient 3a, moderate stenosis), and then further manipulated to increase this value to 71.5% (Patient 3b, severe stenosis). This latter “virtual” scenario was motivated by the clinical context of this patient, where part of the right lung vasculature had been surgically removed in a previous intervention, increasing the likelihood of extensive microvasculature remodeling on the right side. In all cases the pulmonary obstruction values are within the ranges measured by previous clinical studies (Azarian et al., 1997; Miniati et al., 2006). Flow rates in the main pulmonary trunk were obtained from relevant literature and the same inlet flow profile was used in all models (Prakash et al., 2006; Forouzan et al., 2015). From these measures, a physiological velocity profile with ventricular systole from 0 to 380 ms and diastole from 380 to 925 ms was generated and prescribed as inlet boundary condition to the model MPA, as shown in Figure 1E. Two-element Windkessel models were imposed on each of the outlet boundaries of the left and right pulmonary branches (Figure 1E). In each model the vessels walls were considered rigid.

CFD simulations were subsequently carried out using CHeart, a finite-element software platform for personalized cardiovascular simulations, by solving the Navier-Stokes equations for a three-dimensional incompressible flow with a blood density (ρ) of 1,056 kg/m^3^ and a dynamic viscosity (μ) of 3.5 cP (Lee et al., 2016; Hessenthaler et al., 2017). Transient flow simulations were performed on all pre- and post-operative morphologies for comparison of hemodynamic behaviors (Figure 1F). Three cardiac cycles were simulated in each case to achieve a periodic steady-state. The changes in the peak systolic, diastolic, and mean pressure gradients between inlet and outlets, and in the percentages of flow going to the right and left pulmonary arteries were then compared in the pre- and post-operative models to determine the impact of the removal of the proximal obstruction on each patient's hemodynamics. Variations in the WSS magnitude are also presented, whereby the WSS magnitude was calculated based on the tangential component of the traction vector ***t*** = σ***n***, as:

$$\begin{array}{l} WSS=|t-\left(t\cdot n\right)n| \end{array}$$

where **σ** is the Cauchy stress tensor and ***n*** is the normal to the wall. This study was carried out using the High Performance Computing (HPC) facility at King's College London, which comprises a 640 core SGI Altix-UV HPC with Nehalem-EX architecture.

### Windkessel model for the pulmonary microvasculature

The following 2-element Windkessel model was used to model the behavior of the peripheral vasculature on the left and right side (Muthurangu et al., 2005):

(1) $$\begin{array}{r} Q\left(t\right)=\;\frac{p\left(t\right)}{R}+C\;\frac{dp\left(t\right)}{dt} \end{array}$$

where *Q* is the flow rate of blood from the main pulmonary artery, *p* is the blood pressure, *R* the vascular resistance, and *C* the vessel compliance (Muthurangu et al., 2005).

The outlet flow on the LPA and RPA was integrated directly into the Windkessel equation by relating the flow rate *Q*_*i*_ at the relevant boundary Υ_*i*_ to the fluid velocity, υ, i.e.,

(2) $$\int_{\Upsilon_{i}}\upsilon \cdot n_{i}d\Upsilon_{i}=Q_{i}$$

where ***n_i_*** is the normal vector to the boundary plane Υ_*i*_. An estimate based on the ratio between stroke volume and pulse pressure was chosen for the vessel compliance, while the resistance values on the RPA and the LPA were calculated iteratively for each morphology starting with values derived from *in-vivo* measurements (Muthurangu et al., 2005). These initial values were then iteratively tuned using a multi-step procedure to achieve the expected value of the percentage ratio of the stenosed to the non-stenosed pulmonary arterial (i.e., flow ratio) based on the ratios of the areas of the stenosed to the non-stenosed pulmonary arteries (i.e., size ratio) in each patient. First, physiological measurements of the flow splits for varying size ratios were collected from previous studies on patients with branch pulmonary stenosis and a mathematical relationship was subsequently derived by fitting these data using an exponential curve (Sridharan et al., 2006; Ordovás et al., 2007). Second, this function was used to derive the expected flow ratio value given the size ratio in each patient, where the LPA and RPA areas were calculated in the proximal pulmonary branches from the anatomy segmentations. Finally, a sweep study was performed in each case by progressively varying the resistance of the stenosed branch to identify the value that corresponded to the target flow ratio, keeping the vessel compliance fixed. The final values of the resistance in the LPA and RPA of each anatomy are reported in Table 2, together with the degree of stenosis severity and its anatomical location.

**Table 2:** Parameters for the two-element Windkessel model derived for each mesh.

**Patient**	**Diameter reduction (%)**	**Stenosed branch**	Optimized resistance in RPA (Pa^*^s/m^3^)	Optimized resistance in LPA (Pa^*^s/m^3^)
Patient 1	43.8	RPA	677.16E+06	169.29E+06
Patient 2	40.3	LPA	103.80E+06	509.59E+06
Patient 3a	37.1	RPA	152.36E+06	169.29E+06
Patient 3b	71.5	RPA	541.73E+06	169.29E+06

*The vessel compliance was fixed at 1.035E-08 m^3^/Pa*.

### Parametric modeling

Transient flow simulations over multiple cardiac cycles on high-resolution meshes require significant computational resources on HPC facilities, thus limiting the number of patients that can be simultaneously modeled without compromising time efficiency. To improve the models potential for clinical translation, reducing this simulation time is crucial. To achieve the necessary level of time efficiency, an alternative modeling approach based on constant flow simulations was proposed and tested in Patient 2, in both the stenosed and non-stenosed morphologies.

CFD simulations were performed on both stenosed and non-stenosed models with constant values of inlet velocity υ equal to 0.25υ_max_, 0.5υ_max_, and υ_max_ (respectively corresponding to 0.1433, 0.2866, and 0.5732 m/s), where υ_max_ is the maximum inlet velocity of the transient inflow profile. All constant flow simulations were launched in parallel on the HPC facility at King's College London until the solution reached an asymptotic state. The pressure gradient between inlet and outlet, Δ*p*, was then computed in each simulation and a curve was obtained by fitting a 2nd degree polynomial to the data in the parametric space (υ_*n*_, Δ*p*), where the velocity υ_*n*_ represents the constant inflow velocity normalized by υ_max_. The transient pressure gradient Δ*p*(*t*) was finally predicted by substituting the time varying inflow velocity profile υ(*t*) in the polynomial fitting equation, without performing more computationally intensive transient flow simulations. To assess the accuracy of this method, the relative pressure drop obtained from the transient flow simulation was compared to that derived from the polynomial fitting equation using the same inflow velocity profile of the transient flow simulation. Maximum and mean errors between the two pressure curves were calculated in both the stenosed and non-stenosed models for Patient 2.

## Results

Changes in blood flow dynamics, including flow ratio and WSS magnitude, and in the pressure gradients between the stenosed and the non-stenosed model were analyzed in each set of transient flow simulations. The same biomarkers were studied in the constant flow simulations, and in this instance data was extracted from the final time step simulated, representative of an asymptotic state.

### Peak systolic, diastolic, and mean pressure gradients

**Figure 2:**
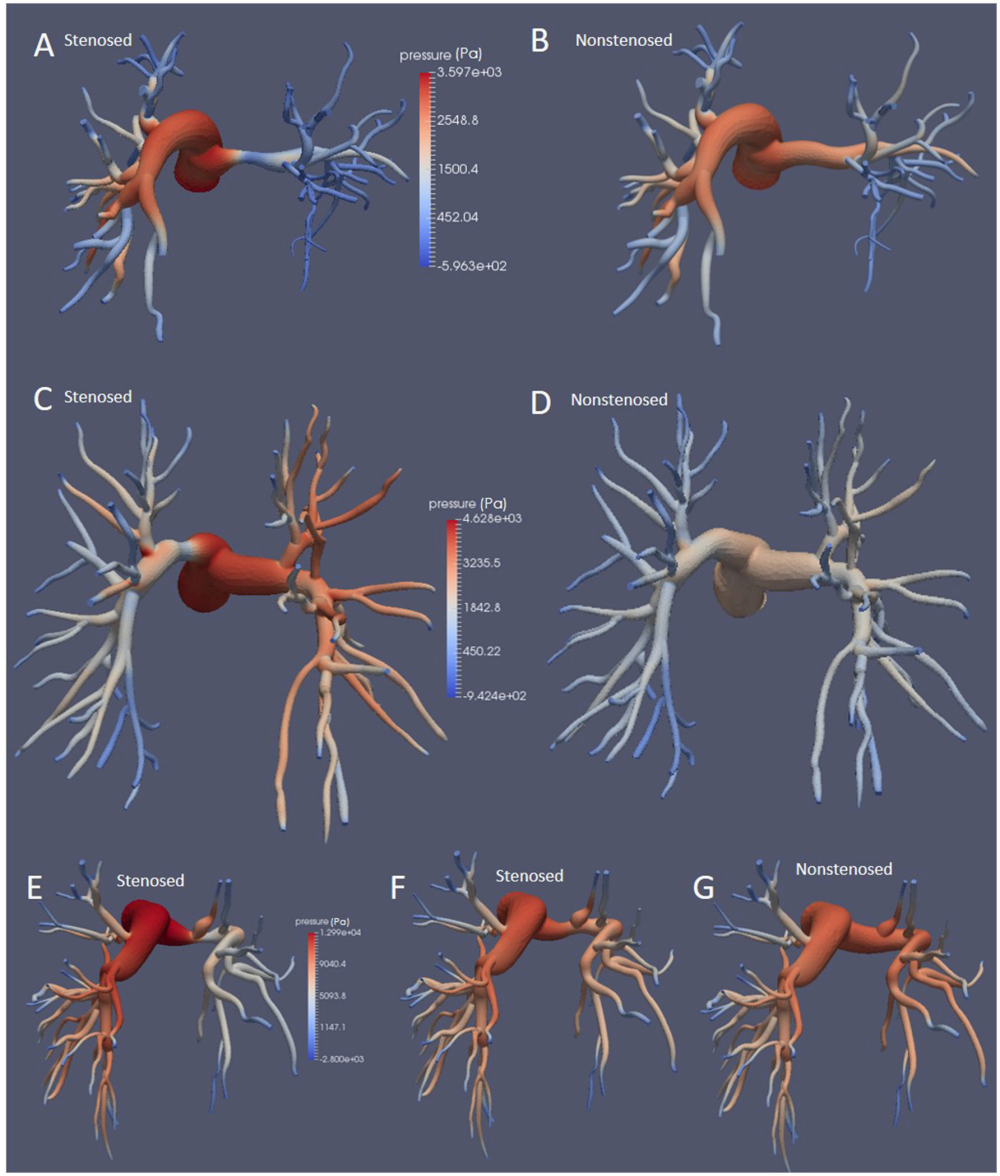
Pressure fields at the wall at peak systole for Patients 1, **(A,B)**, 2 **(C,D)**, and 3 **(E–G)**, in the stenosed and non-stenosed morphologies, posterior view. Patient 3a is illustrated in **(E)** and Patient 3b in **(F)**.

Peak systolic, diastolic, and mean pressure gradients for each morphology are reported in Table 3. As expected, in all patients the stenosed model exhibited higher values in all pressure gradients than the non-stenosed ones. Even though the values reported in Table 3 are calculated based on the pressure transient curves averaged across all outlets, rather than on the LPA and RPA separately, this increase in pressure gradient was driven by the large difference between the inlet and the outlet pressure on the stenosed branch, as shown by the pressure magnitude in Figure 2.

**Table 3:** Pressure gradients between inlet (main pulmonary artery) and outlets (averaged across LPA and RPA) in mmHg for each patient.

	**Stenosed model**			**Non-stenosed model**		
	**DPG**	**Peak systolic PG**	**Mean PG**	**DPG**	**Peak systolic PG**	**Mean PG**
Patient 1	4.73	25.16	4.91	4.29	21.05	4.09
Patient 2	4.10	32.08	5.71	3.41	18.77	3.55
Patient 3a	8.63	61.02	11.07	7.16	57.36	10.31
Patient 3b	9.48	70.45	12.44	7.16	57.36	10.31

*PG, pressure gradient at peak systole (time step 275); DPG, diastolic pressure gradient*.

Overall, upon removal of the thrombotic occlusion, the peak systolic pressure gradient decreased from 25.16 to 21.05 mmHg in Patient 1 (16.3% reduction), from 32.08 to 18.77 mmHg in Patient 2 (41% reduction), and from 61.02 and 70.45 mmHg (in the mild and severe stenosis scenarios, respectively) to 57.36 mmHg in Patient 3, corresponding to a 6.0 and 18.6% reduction. The mean pressure gradients decreased from 4.91 to 4.09 mmHg in Patient 1 and from 5.71 to 3.55 mmHg in Patient 2, indicating a reduction of 16.7 and 37.8%, respectively. In Patient 3 the mean pressure gradient was reduced from 11.07 and 12.44 mmHg, in the moderate and severe stenosis models, to 10.31 mmHg once the stenosis was removed, corresponding to a percentage reduction of 6.9 and 17.1%, respectively. Finally, the DPG was also reduced upon removal of the stenosis. Patients 1 and 2 exhibited a reduction of 9.3% (from 4.73 to 4.29 mmHg) and 16.8% (from 4.10 to 3.41 mmHg), respectively. In Patient 3 the decrease in DPG was 17.0 and 24.5% in the moderate and severe stenosis models (from 8.63 and 9.48 to 7.16 mmHg, respectively).

### Flow split

To appreciate what proportion of the inflow was directed to the stenosed and the non-stenosed branch, the percentage changes in the flow ratio was calculated in both morphologies for each patient and summarized in Table 4. The proportion of flow directed to the formerly stenosed branch increased in all patients upon removal of the stenosis, which resulted in a change in flow ratio ranging from 10 to 60% approximately (Table 4). In Patient 1 the percentage of the total inflow from the main pulmonary artery directed to the stenosed branch (RPA) increased from 22 to 28% upon removal of the occlusion, corresponding to an increase in the flow ratio of 39.3%. In Patient 2 the hemodynamics was more balanced, with ~46% of the total inflow directed to the stenosed branch (LPA); this proportion increased to 48% when the stenosed segment was removed, which corresponded to an increase in the flow ratio of 8.2%. Patient 3 presented a moderate hemodynamic benefit from the removal of the occlusion, with the percentage of inflow to the stenosed branch (RPA) increasing from 28 to 31% (15.4% increase in flow ratio). When the effect of removing the stenosis was investigated in the model with a severe stenosis (Patient 3b), the percentage of inflow to the stenosed branch improved from 22 to 31%, corresponding to a change in flow ratio of 60.7%.

**Table 4:** Flow ratios recorded at peak systole for each patient, and percentage change in flow ratio following removal of stenosis.

	**Flow ratio in stenosed model**	**Flow ratio in non-stenosed model**	**% flow ratio change**
Patient 1	0.28	0.39	39.3
Patient 2	0.86	0.92	8.2
Patient 3			
3a (mild stenosis)	0.39	0.45	15.4
3b (severe stenosis)	0.28	0.45	60.7

### Blood flow dynamics and wall shear stress

**Figure 3:**
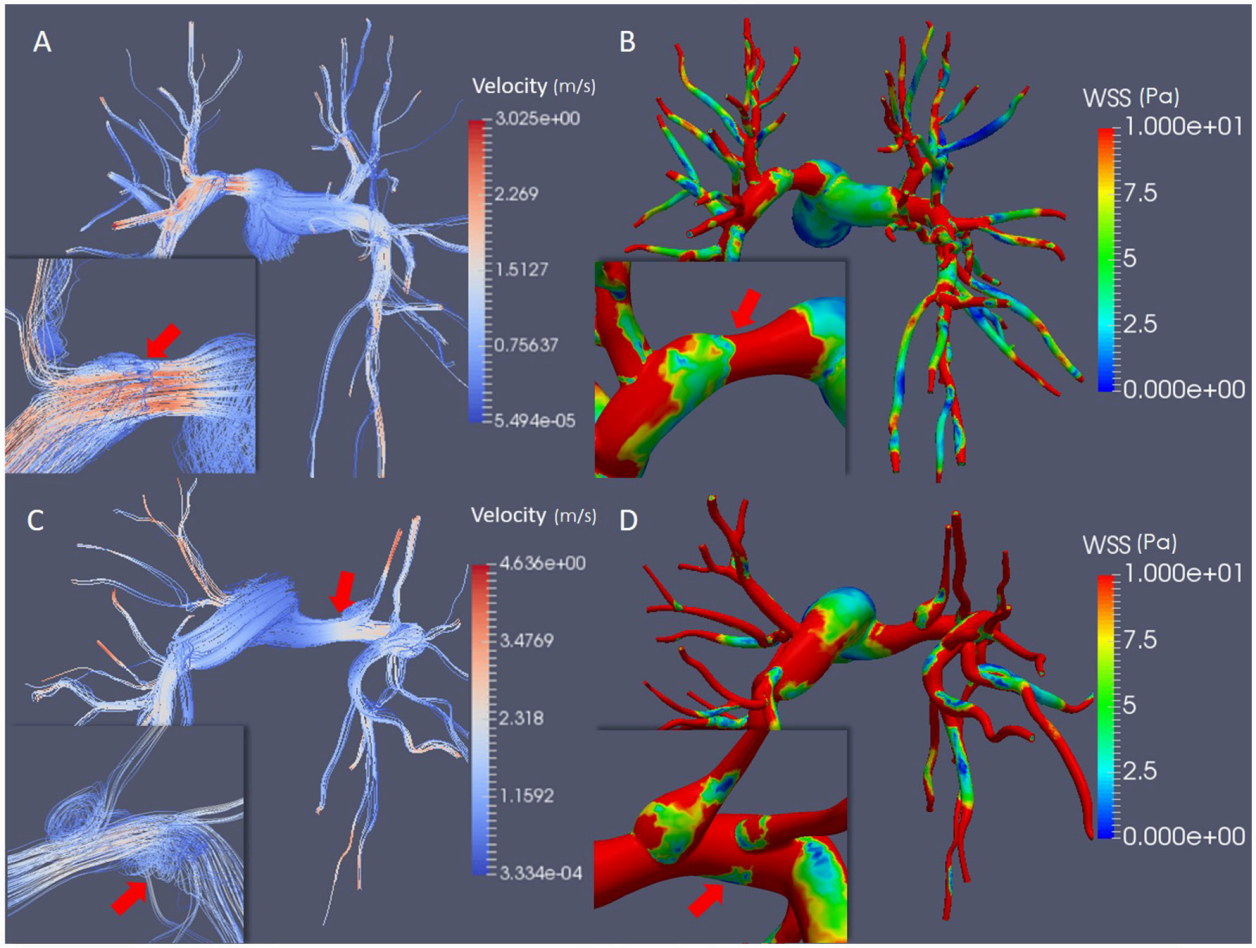
Simulation results at peak systole. **(A)** Streamlines colored by velocity magnitude in Patient 2. **(B)** WSS magnitude in Patient 2. **(C)** Streamlines colored by velocity magnitude in Patient 3a; **(D)** WSS magnitude in Patient 3a. The mild stenosis case was employed for Patient 3a. The inserts show the flow behavior and WSS magnitude in the stenosed segment of the pulmonary arteries, highlighting the flow recirculation regions, and the helical flow **(A,C)** and the drop in the WSS magnitude **(B,D)**.

The blood flow velocity field and the distribution of WSS magnitude were also analyzed and compared between the models. The stenosed models of Patients 2 and Patient 3a were chosen for comparison in Figure 3.

Patient 2 exhibited blood flow velocities of up to 3.025 m/s at peak systole in the stenosed segment, where the WSS peaked at 31 Pa before dropping to 2 Pa downstream of the stenosis (Figures 3A,B). Small areas of slow recirculating blood flow are visible downstream of the stenosis, where the WSS magnitude decreased abruptly (Figure 3A, insert).

In Patient 3 the peak systolic blood flow velocity in the stenosed segment reached 4.636 m/s, which corresponded to an increase in WSS magnitude to 33 Pa (Figures 3C,D). Unlike Patient 2, in this case dilated regions are observed downstream of the occlusion and in the secondary branch originating from the stenotic segment, where the magnitude of the WSS dropped to <1 Pa (Figure 3D, insert). A large region of recirculating blood flow and a low velocity helical flow developed in these regions and was associated with low WSS magnitude (Figures 3C,D, insert).

### Constant flow simulations and parametric modeling

**Figure 4:**
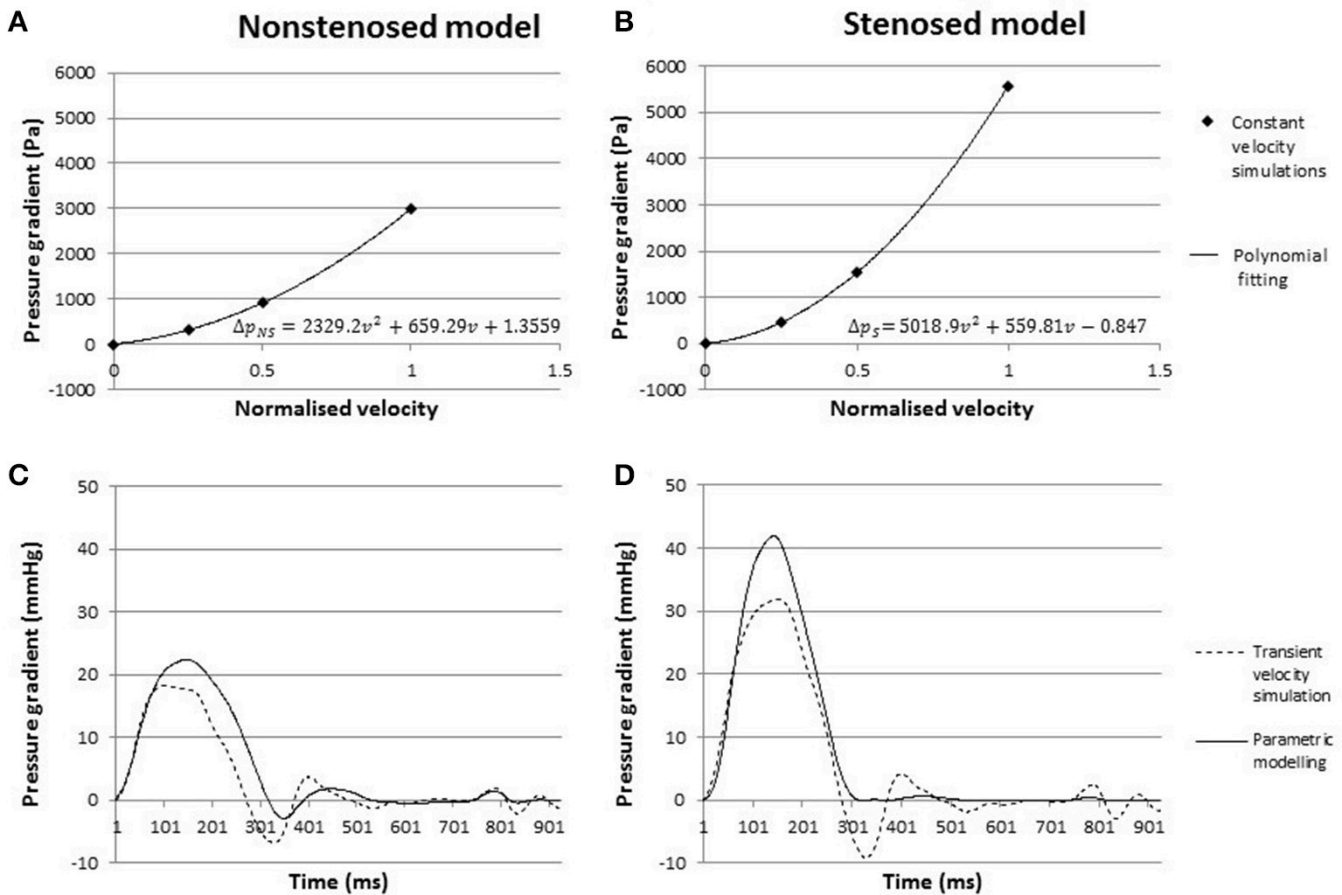
**(A,B)** Pressure gradients from constant flow simulations in the non-stenosed **(A)** and stenosed model **(B)**, with fitted parametric curve. **(C,D)** Pressure gradient over time in the non-stenosed **(C)** and stenosed **(D)** model. The solid line indicates the pressure gradient predicted by parametric modeling, while the dashed line represents an average of the gradients obtained in LPA and RPA from the transient flow simulation.

Parametric modeling was then employed to investigate whether one of the biomarkers of interest, pressure gradients, could be calculated from discrete constant flow simulations. The pressure gradient for each of the flow simulations with constant inflow velocity was recorded in the stenosed and the non-stenosed models of Patient 2. The polynomial fitting equations for the stenosed and non-stenosed cases are reported in Figures 4A,B. The corresponding predicted curves of transient pressure gradients in the stenosed and non-stenosed model, Δ*p*_*S*_(*t*) and Δ*p*_*NS*_(*t*), respectively, were compared to the data from the transient flow simulations with the same prescribed inflow velocity profile in Figures 4C,D.

In the non-stenosed model, the maximum pressure gradient at peak systole predicted using the constant flow approach was 22.4 mmHg, which was 19% higher than the peak systolic pressure gradient derived from the transient flow simulations. In the stenosed mesh, the parametric modeling predicted a peak systolic gradient of 41.8 mmHg, which compared to the transient flow simulation peak resulted in an error of ~30%. The constant flow approach overestimated the mean pressure gradient by 24 and 47% in the stenosed and non-stenosed models, respectively. Overall the mean absolute error over the whole cycle is 1.51 mmHg for the stenosed mesh, and 1.64 mmHg in the non-stenosed mesh, derived by subtracting the two curves in time. For both models, for 75% of the cycle, the absolute value of the difference is below 5.5 mmHg. These results were obtained without modifying the specific memory requirements in one third of the computational time of the transient flow simulations on the same HPC cluster, thus making the parametric modeling approach less computationally intensive than the transient flow simulations.

## Discussion

This study provides a proof of concept on how high performance computing, imaging data and numerical modeling can be successfully integrated to address the specific clinical questions posed by CTEPH. We performed patient-specific CFD simulations on realistic models of the pulmonary arteries in patients affected by CTEPH to help inform patient selection criteria for surgical intervention by pulmonary thromboendarterectomy. The additional information provided by the models is particularly relevant for patient management in CTEPH, which is an under-diagnosed progressive disease whose stage at the point of intervention can vary significantly from patient to patient. The success of surgery depends on the degree of peripheral vascular remodeling that has occurred in the lungs since the formation of the thrombotic occlusion. Our approach allows to model the contribution of the occlusion (proximal resistance) and of the pulmonary vasculature remodeling (peripheral resistance) to the overall pulmonary vascular resistance. By quantifying changes in clinical biomarkers such as pressure gradients, WSS and flow balance between LPA and RPA, this technique can help assessing the hemodynamic effects introduced by the removal of a proximal occlusion in each patient. Such information is very challenging to obtain *in-vivo*, making pre-operative patient selection one of the most problematic issues of CTEPH management. By establishing if the main cause of the disease in each individual is proximal or peripheral, our personalized models can provide potentially decisive data to inform treatment.

### Metrics for patient selection: pressure gradients and flow ratio

In CTEPH, pressure gradients and flow ratio between RPA and LPA provide extremely valuable information for characterizing the disease progression. However, pressure and flow cannot be directly quantified from standard imaging data, and even when this assessment can be performed, e.g., using advanced flow imaging techniques such as PC-MRI, its accuracy is often hampered by low spatio-temporal accuracy. While the gold standard for pressure and flow measurements is cardiac catheterization, this technique is highly invasive and only allows for measurement at discrete proximal locations in the pulmonary trunk. Numerical flow simulations present the advantage of providing noninvasive measurements for all points in the morphology of interest, including peripheral vessels.

Results showed that the removal of a proximal occlusion in the left or right pulmonary artery led to a successful reduction of the pressure gradients between the main pulmonary artery and the peripheral vasculature in Patients 1 and 2, while in Patient 3 the reduction was less significant. In this case, a pressure gradient reduction similar to that of Patent 1 (16.9%) could be achieved only when a more severe stenosis was introduced. This suggests that in Patient 3 the degree of remodeling in the peripheral vasculature represents a relatively larger contribution to the overall pressure gradients than the localized proximal resistance due to the thrombotic occlusion, thus implying that the surgical removal of the stenosis might have a less beneficial effect in this case than for Patients 1 and 2.

The removal of the stenosis also changed the flow ratio between right and left pulmonary arteries, increasing the flow rate to the repaired branch. While Patients 1 and 3a had a similar reduction in diameter, removal of the segment resulted in different levels of improvements in flow balance. The percentage change in flow ratio was higher in Patient 1 (39.3%) than in Patients 2 and 3a, where it reached 8.2 and 15.4%, respectively. The small variation observed in Patient 2 can be related to the fact that in this case the flow ratio is close to the values found in normal subjects (Cheng et al., 2005). This suggests that for Patient 1 the removal of the proximal occlusion was more beneficial to the balance of pulmonary flow than in Patients 2 and 3. For this latter case, only when a severe stenosis was introduced in the model and subsequently removed, the flow ratio increased significantly by more than 60%. This result is in agreement with the hypothesis that removal of the stenosis is less beneficial in this case than in the other two. It is worth noticing that this patient is the only one where the DPG value was above the critical threshold for peripheral remodeling of 7 mmHg.

### Constant flow simulations

The estimation of transient pressure gradients from constant flow simulations provided a computationally efficient method for the assessment of the peak systolic and mean pressure gradient.

During diastole, when changes in the inlet velocity magnitude are very small, the pressure gradient curve from the constant flow simulation was in agreement with the simulation results using the time-varying inflow velocity profile. However, parametric modeling resulted in significant differences in the peak systolic pressure gradients, with a 30% overestimation compared to the transient inflow simulation result in the stenosed model. Such overestimation is present in the non-stenosed case as well, albeit of smaller magnitude (20%). When the percentage of improvement in the peak systolic pressure gradient following the removal of the stenosis is considered, however, both models provide a similar result: the peak systolic pressure gradient was reduced by ~41% according to the transient inflow simulation and by 46% in the results from parametric modeling.

Overall, parametric modeling provides an effective strategy to reduce computation time and to estimate the expected change in peak systolic pressure gradients post-operatively, albeit the peak magnitude of the pressure gradient in each model is overestimated by this simplified approach. While each transient flow simulation took just under 5 h to complete, all three constant flow simulations were launched at the same time and required only 1 h of computations, effectively reducing the simulation time. This is particularly relevant in clinical applications, where the prompt availability of investigation is essential for an effective clinical translation of the modeling results.

### Wall shear stress

WSS is a biomarker that cannot be derived from standard anatomical imaging data such as the CTPA scans used in this study. Non-routine imaging techniques, such as PC-MRI, are usually needed in order to reconstruct flow dynamics in time and space, from which WSS can be calculated: however these type of imaging data requires longer acquisition times to achieve the necessary spatio-temporal resolution for an accurate estimation of the WSS. WSS nevertheless is a significant metric in the context of vascular pathology. It is well-known that endothelial cells, when exposed to normal shear forces, produce agents with antithrombotic properties, but when the wall shear stress is outside of the normal ranges this mechanism is disrupted and pathologies such as arteriosclerosis and thrombogenesis can develop (Tang et al., 2012). Low wall shear stress is linked to vasodilation, aneurysm formation, and the development of atherosclerotic plaques (Jiang et al., 1999; Boussel et al., 2008). In patients affected by pulmonary hypertension, WSS is decreased in the pulmonary arteries, a phenomenon which is associated with vasodilation, increased cardiac output, and a subsequent decrease in pulmonary vascular resistance. The compensatory effect resulting from vessel dilation can initially counter the disease progression, however drug therapy based on epoprostenol is necessary to maintain a long-term reduction in pulmonary vascular resistance that can stabilize the disease, albeit without reversing its progression (McLaughlin et al., 1998). Such a mechanism can explain the results observed in Patient 3, where the magnitude of WSS downstream of the stenosis was lower than in Patient 2 despite a similar diameter reduction in the stenotic segment, but a much more dilated wall was observed in the same region. The lower average magnitude of WSS in Patient 3 could also be associated with hypertension, which was suggested by a DPG value greater than 7 mmHg.

Understanding the relationship between low WSS magnitude, vessel dilation and PVR reduction can reveal key information on the progression of pulmonary hypertension and the degree of remodeling in the vasculature. Therefore, WSS may prove an insightful biomarker to risk-stratify patients based on hemodynamic features. Despite its importance, WSS calculations are often not available to the clinician due to the difficulty in reliably deriving this biomarker from imaging data alone. In this context, CFD simulations can provide valuable insight into patient assessment by quantifying the WSS magnitude and its changes over time.

### Limitations

Simulations for this study were based on a number of assumptions. We prescribed that arterial walls were rigid, and expressed the behavior of the peripheral vasculature below subsegmental level using a lumped parameter model, i.e., a two-element Windkessel model. The anatomical segmentation, even if performed using a semiautomatic technique, could only be carried out up to a limited degree of detail. The high spatial and contrast resolution of dual energy CT acquisitions allow the visualization of small diameter arterioles, making it possible to trace pulmonary vasculature well beyond subsegmental level. A cut-off generation or caliber beyond which no vessel is segmented was defined since tracing every individual terminal branch would become excessively time consuming and therefore unfeasible, particularly if considering potential clinical applications. Besides being an obvious trade-off between anatomical accuracy and operational efficiency, defining such endpoint is a complex task in itself, with no supporting literature available to guide the decision. Overall, therefore, segmentation of the pulmonary arteries is highly affected by operator variability, thus limiting the reproducibility of the experiment. Future work to improve this limitation could include development of fully automatic segmentation techniques, like statistical shape models or atlas-based approaches (Shikata et al., 2004; Buelow et al., 2005).

The models in this pilot study were also based on post-operative scans due to unavailability of pre-operative datasets, and the occlusion level and position was idealized based on the clinical history of the patients. However, the aim of this preliminary work was to define the methodology and technical feasibility of the study and this limitation will be overcome in future studies using pre-operative imaging acquisition protocols. Similarly, due to limited availability of pre-surgical clinical records for the patients examined, indexes and comparative data for validation, e.g., comparable pressure and velocity fields, have been chosen from relevant literature. Specifically, in all patients the same idealized inflow velocity profile was employed in both the stenosed and the non-stenosed models, while evidence suggests that in CTEPH patients waveform diverges from the standard and changes markedly between patients (Kim, 2006). Rather than a methodological shortcoming, this limitation is down to incomplete clinical datasets and thus can be addressed in future studies by prospectively acquiring Color Doppler ultrasound data in addition to morphological CTPA scans.

## Conclusion

This study shows that patient-specific biophysical modeling of pulmonary vasculature has a potential role in optimizing CTEPH patient selection for PTE and potentially become an effective tool for a quantitative classification of CTEPH types and treatment in the longer term. Specifically, providing a quantitative prediction of the changes in pressure gradients in the pulmonary tree and flow ratio between the RPA and LPA can help identify patients in which chronic hypertension is mostly due to peripheral remodeling, and therefore is not significantly ameliorated by removal of a thrombotic occlusion. Our results show that the improvement in both pressure gradients and flow balance is different in patients with similar diameter reduction in the stenotic segment, implying that such assessment goes beyond the simple evaluation of the percentage of stenosis present in the vasculature, which provides a purely anatomical criterion for intervention. Linking the blood flow dynamics to the patient morphology is thus a key step to determine whether the hemodynamic benefits of the stenosis removal are sufficiently significant to justify surgical intervention.

In addition to this application to CTEPH, this approach is highly flexible and can be generalized to perform individualized assessment of any disease characterized by a high degree of morphological variability. Thanks to increasingly powerful HPC resources, this additional information on the pathophysiological mechanisms linking altered hemodynamics and disease progression can now be computed in a timeframe compatible with clinical needs, which represents a major step forward in the clinical translation of mathematical modeling. To further address this issue, we also presented a time-efficient approach based on constant flow simulations and parametric curve fitting that has the ability to reproduce transient pressure gradients for a given inflow velocity profile, thus reducing computational demand and optimizing the usage of HPC resources. By addressing a specific clinical question, this study provides a proof of concept that mathematical modeling combined with high performance parallel computing holds significant potential for assisting the clinical decision-making process for CTEPH patients who are potential candidates to PTE.

## Author contributions

AdV together with DN supervised the project from beginning to end and coordinated the authors' efforts, performing the simulations, and working on the data analysis. MS worked on the project producing the meshes on which simulations were run, as well as contributing to the analysis of results, and together with AdV authored the manuscript. PS and JD were responsible for patient recruitment and acquisition of the data employed to perform this study.

### Conflict of interest statement

The authors declare that the research was conducted in the absence of any commercial or financial relationships that could be construed as a potential conflict of interest.
